# On the Maximum Storage Capacity of the Hopfield Model

**DOI:** 10.3389/fncom.2016.00144

**Published:** 2017-01-10

**Authors:** Viola Folli, Marco Leonetti, Giancarlo Ruocco

**Affiliations:** ^1^Center for Life Nanoscience, Istituto Italiano di TecnologiaRome, Italy; ^2^Department of Physics, Sapienza University of RomeRome, Italy

**Keywords:** maximum storage memory, feed-forward structure, random recurrent network, Hopfield model, retrieval error

## Abstract

Recurrent neural networks (RNN) have traditionally been of great interest for their capacity to store memories. In past years, several works have been devoted to determine the maximum storage capacity of RNN, especially for the case of the Hopfield network, the most popular kind of RNN. Analyzing the thermodynamic limit of the statistical properties of the Hamiltonian corresponding to the Hopfield neural network, it has been shown in the literature that the retrieval errors diverge when the number of stored memory patterns (*P*) exceeds a fraction (≈ 14%) of the network size *N*. In this paper, we study the storage performance of a generalized Hopfield model, where the diagonal elements of the connection matrix are allowed to be different from zero. We investigate this model at finite *N*. We give an analytical expression for the number of retrieval errors and show that, by increasing the number of stored patterns over a certain threshold, the errors start to decrease and reach values below unit for *P* ≫ *N*. We demonstrate that the strongest trade-off between efficiency and effectiveness relies on the number of patterns (*P*) that are stored in the network by appropriately fixing the connection weights. When *P*≫*N* and the diagonal elements of the adjacency matrix are not forced to be zero, the optimal storage capacity is obtained with a number of stored memories much larger than previously reported. This theory paves the way to the design of RNN with high storage capacity and able to retrieve the desired pattern without distortions.

## 1. Introduction

A vast amount of literature deals with neural networks, both as model for brain functioning (Amit, 1989), and as smart artificial systems for practical applications in computation and information handling (Haykin, 1999).

Among the different possible applications of artificial neural networks, those referred to as “associative memory” are particularly important (Rojas, 1996), i.e., circuits with the capability to store and retrieve specific information patterns. According to Amit et al. (1985a,b) there is a natural limit for the usage of an *N* nodes neural network built according to the Hebbian principle (Hebb, 1949) as associative memory. The association is embedded within the connection matrix which has a dyadic form: the weight connecting neuron *i* to neuron *j* is the product of the respective signals. The limit of storage is linear with *N*: an attempt to store a number *P* of memory elements larger than α_*c*_*N*, with α_*c*_ ≈ 0.14, results in a “divergent” (order *P*) number of retrieval errors. In order to be effective (low retrieval error probability) a neural network working as associative memory cannot be efficient (i.e., it can store only a small number of memory elements). This is particularly frustrating in practical applications, as it strongly limits the use of artificial neural networks for information storage, especially since it is well known that the number of fixed points in randomly connected (symmetric) neural networks shows an exponential relation with *N* (Tanaka and Edwards, 1980; Sompolinsky et al., 1988; Wainrib and Touboul, 2013).

Contemporaneous to Amit et al., Abu-Mostafa, and St. Jaques (Abu-Mostafa et al., 1985) claimed that the number of fixed points that can be used for memory storage in a Hopfield model with a generic coupling matrix is limited to *N* (i.e., *P*<*N*). Soon after, Mc Eliece et al. (1987), considering only the Hebbian dyadic form for the coupling matrix, found a more severe limitation: the maximum *P* scales as *N*/log(*N*). In a more recent study, Sollacher et al. (2009) designed a network of specific topology, reaching α_*c*_-values larger than 0.14, but still maintaining the limit of a linear *N* dependence of the maximum storage capacity. The storage problem remains an open research question (Brunel, 2016).

In this letter we show that the existence of a critical *P*/*N*- value in the Hebbian scheme for the coupling matrix is only part of the story. As demonstrated in Amit et al. (1985a,b), the limit *P*<α_*c*_*N* holds in the region where *P*<*N*. In all previous studies, the diagonal elements are removed from the dyadic form of the coupling matrix. Here we show the existence of a not yet explored region in the parameter space, with *P*≫*N*, where the number of retrieval errors decreases with increasing *P* and reaches values lower than one. This region can be found by not removing the diagonal elements. Strictly speaking the present model is not a “Hopfield model,” as in the latter case the diagonal elements are forced to vanish and—as we will see- bring significant differences in the network behavior. In order to avoid confusion, let us call the present model as “Hopfield model with autapses” or “Generalized Hopfield model.” This strategy allows the design of effective and efficient associative memories based on artificial neural networks. In the following we will derive analytically the probability of retrieval errors, validate these results by their comparison with a numerical simulation and study the efficiency of the system as a function of *P* and *N*.

## 2. Methods

### 2.1. Network model

In an artificial neural network working as associative memory, one deals with a network of *N* neurons of which each one has state *s*_*i*_ (*i* = 1…*N*) that can be “active” (*s*_*i*_ = 1) or “quiescent” (*s*_*i*_ = −1). The configuration of the whole network is given by the vector $\bar{s}\equiv \{s_{1},s_{2},..s_{N}\}$ and its temporal evolution follows the parallel non-linear dynamics:

(1) $$s_{i}(t+\Delta t)=E[s_{i}(t)]≐\text{sign}\left[\sum_{j=1}^{N}J_{ij}s_{j}(t)\right],\;$$

where **J** = {*J*_*ij*_} is the connection matrix. We set external inputs to be equal to 0. We assume a symmetric bimodal distribution for the synaptic polarities in the wiring matrix **J**, so 50% of the connections are excitatory and 50% inhibitory. After a transient time related to the finite value of *N*, the network reaches a fixed point, *s*_*i*_ = *E*[*s*_*i*_], or a limit cycle of length *L*, s_i_ = *E*^(*L*)^[*s_i_*].

### 2.2. The hebbian rule and the storage memory

Previous work has studied the cycle length and transient time distribution as a function of the properties of **J** (Gutfreundt et al., 1988; Sompolinsky et al., 1988; Derrida, 1989; Bastolla et al., 1997). In order to work as an associative memory, the matrix **J** must be tailored in such a way that one or more patterns of neurons are fixed points of the dynamics in Equation (1), i.e., they are the “memory elements” stored in the network. To store one pattern $\bar{\xi }$, the connection matrix is simply the dyadic form given by *J*_*ij*_ = ξ_*i*_ξ_*j*_ ^1^ , while to store a generic number *P* of patterns ${\bar{\xi }}^{\mu }$ (μ = 1…*P*) one follows the storage prescription of Cooper (1973) and Cooper et al. (1979), who exploited an old idea which goes back to Hebb (1949) and Eccles (1953) and which states that the change in synaptic transmission is proportional to the product of the signals of pre and post-synaptic neurons. The process for which each matrix element is appropriately determined is called *learning*. Specifically, the “Hebbian” rule results in the following expression for the connectivity matrix,

(2) $$J_{ij}=\frac{1}{P}\sum_{\mu =1}^{P}\xi_{i}^{\mu }\xi_{j}^{\mu }.\;$$

The set of vectors ${\bar{\xi }}^{m}u$ is known as “training set.” In this case, it is not guaranteed that each ${\bar{\xi }}^{\mu }$ is a fixed point. In other words, ${\bar{\xi }}^{\mu }$ is stable in probabilistic sense. Further, the probability for ${\bar{\xi }}^{\mu }$ to be a fixed point depends on the values of *P* and *N*. This dependence has been first studied by Hopfield (1982); Hopfield et al. (1983); Hopfield (1984) who concluded that the retrieval of the memory stored in the Hebbian matrices is guaranteed up to a *P*-value which is a critical fraction on the number of network nodes *N* of the order of 10–20%. Above this value, the associative memory quickly degrades. Following these studies, Amit et al. (1985a,b), who noticed the similarity between the Hopfield model for the associative memory and the spin glasses, developed a statistical theory for the determination of the critical *P*/*N* ratio, that turned out to be ≈ 0.14, in good agreement with the previous Hopfield estimation. Above *P*=0.14*N* the number of errors is so large that the network based on the Hebbian matrix is no longer capable to work as an associative memory. All these studies assumed a modified form of Equation (2): the diagonal elements of **J** are forced to be zero.

### 2.3. Numerical simulations and data analysis

In order to demonstrate the validity of our analytic results (see Section 3), we perform numerical simulations by evolving the network model as described in Equation (1). We design the default network by fixing the NxN recurrent connections as given in Equation (2), by randomly assigning the value ±1 to $\xi_{i}^{\mu }$ and retaining the diagonal elements. So, the *N*(*N*−1) connections are 50% excitatory and 50% inhibitory and the *N* neurons can form self-connections. We then run simulations by varying the size of the network, *N* = 50, .., 200 and the number of stored memories in Equation (2), *P* = 1, …, 2000. Finally, for each pair of *N* and *P*, we perform 1000 different random realizations.

All *P* patterns introduced in Equation (2) are given as input to the network and their dynamics is followed until the network reaches the equilibrium state. The initial patterns are chosen among those that were stored in the adjacency matrix and that have been randomly chosen in the designing of the network. Evolved patterns were recorded at each time step and compared with the initial one. Then, if the evolved pattern is different from the initial state, we calculated the temporal evolution of the distortion (number of wrong bits) and determined the probability that one of the bits was wrong, the probability that the whole vector was exactly recovered, and the number of memory patterns that could not be recovered, as a function of *N* and *P*. Basically, to calculate the storage capacity, it is sufficient to determine all these quantities by using the distortion between the stimulus (the stored memory) and its first evolved pattern.

## 3. Results

### 3.1. The probability of recovery

In order to investigate the maximum storage memory of our model, we calculate the one-step dynamical evolution. We give as input a vector of the training set and we calculate a single step of the dynamical evolution according to Equation (1). Then, we compare the output with the input. We aim to look whether or not a vector, ${\bar{\xi }}^{\mu }$, belonging to the training set, is truly a fixed point. If ${\bar{\xi }}^{\mu }$ is a fixed point, the output coincides with the input, and the recovery has been successful. If ${\bar{\xi }}^{\mu }$ is not a fixed point, the two vectors differ for at least a single bit. We now derive an analytical expression for the probability that the recovery of a stored pattern was not successful. The first step is to find the probability *p*_*B*_ that -given the matrix **J** of Equation (2)- a single element of the vector (a “bit”) was wrong, i.e., the probability that $E\left[\xi_{i}^{\mu }\right]\neq \xi_{i}^{\mu }$. Basically, we need to evolve a vector ${\bar{\xi }}^{m}u$ (from the training set) for one step and count how many bits of its time evolution are different from the bits of ${\bar{\xi }}^{m}u$ itself. Obviously, if ${\bar{\xi }}^{m}u$ actually is a fixed point, this distance vanishes. On the contrary, ${\bar{\xi }}^{m}u$ is not a fixed point, the network has made a recovery error. Thus, *p*_*B*_ (or better, *p*_*V*_, as we see in the next paragraph) measures “how many” training set vectors are not fixed points. The argument of the *sign* function in Equation (1) is $A_{i}^{\mu }=\overset{N}{\underset{j=1}{\sum }}\overset{P}{\underset{\nu =1}{\sum }}\xi_{i}^{\nu }\xi_{j}^{\nu }\xi_{j}^{\mu }$, this contains *NP* terms among which there are *N*+*P*−1 terms (those with *j*=*i* ^2^ and those with ν=μ) where two out of the three ξ of the product are equals to each other $\xi_{i}^{\nu }$ and the third is $\xi_{i}^{\mu }$. Thus $A_{i}^{\mu }=(N+P-1)\xi_{i}^{\mu }+T_{i}^{\mu }$, with $T_{i}^{\mu }=\overset{N}{\underset{j\neq i}{\sum }}\overset{P}{\underset{\nu \neq \mu }{\sum }}\xi_{i}^{\nu }\xi_{j}^{\nu }\xi_{j}^{\mu }$. The first term is the “coherent” one, its sign is identical to $\xi_{i}^{\mu }$, and it will -if dominant- guarantee that ${\bar{\xi }}^{\mu }$ is a fixed point of the dynamics. The second term $T_{i}^{\mu }$ , on the contrary, is “noise” and its presence can either reinforce or weaken the stability of $\xi_{i}^{\mu }$ as fixed point. Specifically, if $\left|T_{i}^{\mu }|\;>(N+P-1\right)$ and sign$\left(T_{i}^{\mu }\right)\neq \xi_{i}^{\mu }$, then the *i*-*th* bit of the vector ${\bar{\xi }}^{\mu }$ will turn out to be wrong. The quantity $T_{i}^{\mu }$ is the sum of (*N*−1)(*P*−1) statistically independent terms, each one being +1 or −1. Therefore, for large enough *P* and *N*, its distribution *N*(*T*) can be approximated by a gaussian with zero mean and standard deviation $\sqrt{\left(N-1\right)\left(P-1\right)}$:

(3) $$N(T)=\frac{e^{-T^{2}/(2(N-1)(P-1))}}{\sqrt{2\pi (N\text{​}-\text{​}1)(P\text{​}-\text{​}1)}}.\;$$

It is now straightforward to determine the probability that $\left|T_{i}^{\mu }|\;>(N+P-1\right)$ and sign$\left(T_{i}^{\mu }\right)\neq \xi_{i}^{\mu }$, thus that one of the bits of $E\left[\xi_{i}^{\mu }\right]$ was wrong, as $p_{B}=\int_{N+P-1}^{\infty }dTN\left(T\right)$. In conclusion:

(4) $$p_{B}=\frac{1}{2}\left[1-\text{erf}\left(\frac{N\text{​}+\text{​}P\text{​}-\text{​}1}{\sqrt{2(N\text{​}-\text{​}1)(P\text{​}-\text{​}1)}}\right)\right].\;$$

**Figure 1:**
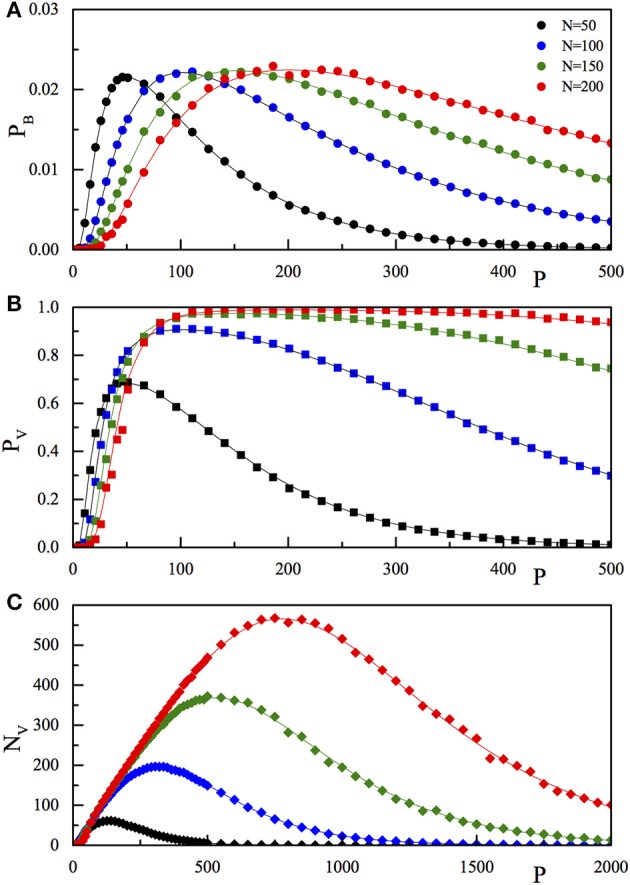
**Comparison of the results of the numerical simulation (***p***_***B***_, full dots A**; *p*_*V*_, full squares, **B**; *N*_*V*_, full diamonds, **C**) with the corresponding theoretical function (*p*_*B*_, Equation 4; *p*_*V*_, Equation 5; *N*_*V*_, Equation 6) reported as full lines. The three quantities are reported as a function of *P* for fixed *N*. The values of *N* are 50 (black), 100 (blue), 150 (green), and 200 (red). The *P* range in **(C)** is extended with respect to **(A,B)**.

**Figure 2:**
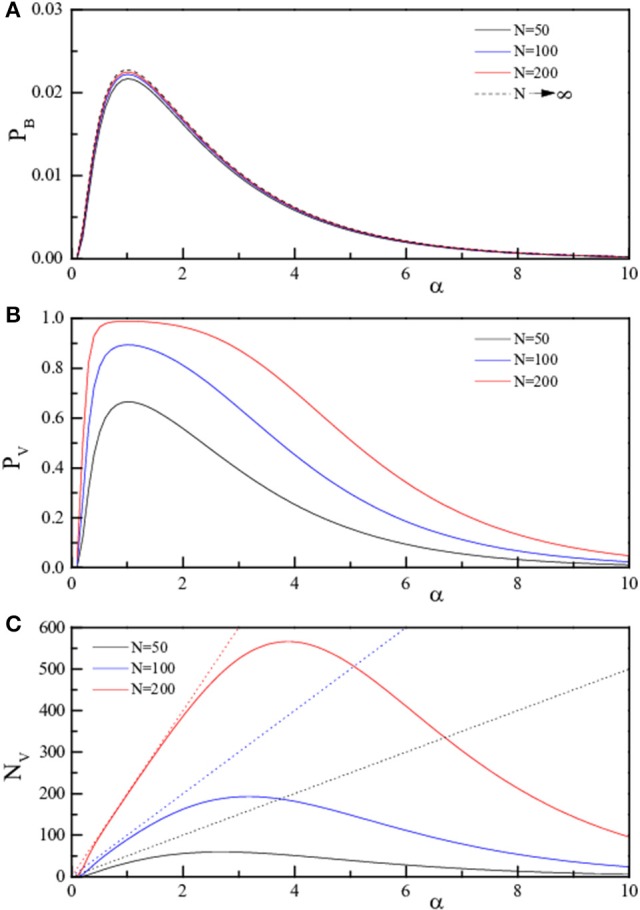
**Theoretical curves for the three quantities ***p***_***B***_, ***p***_***V***_, and ***N***_***V***_ (***p***_***B***_, Equation 4, A**; *p*_*V*_, Equation 5, **B**; *N*_*V*_, Equation 6, **C**) reported as full lines. The three quantities are reported in linear scale as a function of α = *P*/*N* for fixed *N*. The values of *N* are 50 (black), 100 (blue), and 200 (red). The dotted lines in **(C)** represent *N*_*V*_ = *P*.

It is worth to note that this expression is symmetric under the exchange of *P* with *N*, and that for large *P* and *N*, with *P*/*N* = 1, it tends to $(1-erf(\sqrt{2}))/2\approx$0.02275 which corresponds to the maximum of probability in a wrong recovery of a single bit (see Figures 1, 2).

The second step is the determination of the probability *p*_*V*_ that one of the P vectors encoded into the connection matrix (the training set) turns out not be a fixed point. If only a single bit of the vector is wrong, the whole vector is considered “wrong.” Since there are *N* bits that can be wrong, the probability *p*_*V*_ will be much higher than *p*_*B*_. The calculation is straightforward, in order not to be wrong, all the bits of the vector ${\bar{\xi }}^{\mu }$ must be right, thus *p*_*V*_ = 1−${\left(1-p_{B}\right)}^{N}$, therefore:

(5) $$p_{V}=1-{\left[\frac{1}{2}+\frac{1}{2}\text{erf}\left(\frac{N\text{​}+\text{​}P\text{​}-\text{​}1}{\sqrt{2(N\text{​}-\text{​}1)(P\text{​}-\text{​}1)}}\right)\right]}^{N}.\;$$

Finally, the number, *N*_*V*_, of memory vectors that are not recovered, i.e., that are not true fixed points of the dynamics is given by *Pp*_*V*_, that is:

(6) $$N_{V}=\left[1-{\left[\frac{1}{2}+\frac{1}{2}\text{erf}\left(\frac{N\text{​}+\text{​}P\text{​}-\text{​}1}{\sqrt{2(N\text{​}-\text{​}1)(P\text{​}-\text{​}1)}}\right)\right]}^{N}\right]P$$

### 3.2. The asymptotical approximation

Equations (4), (5) and, in particular, Equation (6) represent the main result of this work. Before showing their validity, via a comparison with numerical simulations, and discussing their relevance in the framework of artificial neural networks, it is important to present the asymptotical approximation for *N*_*V*_. The argument of the error function, for either *P*≫*N* or *P*≪*N*, is large, and can be expanded as erf(*x*) ≈ 1−exp$\left(-x^{2}\right)/\left(x\sqrt{\pi }\right)$. Furthermore, as *p*_*B*_ is exponentially small with *N* (or *P*) for large *N* (*P*), we use, ${\left(1-p_{B}\right)}^{N}\approx \left(1-Np_{B}\right)$. Thus, for large *N* or large *P*:

(7) $$p_{V}\approx \frac{N^{3/2}P^{1/2}e^{-\frac{{\left(N+P\right)}^{2}}{2NP}}}{\sqrt{2\pi }(N+P)}$$

(8) $$N_{V}\approx \frac{N^{3/2}P^{3/2}e^{-\frac{{\left(N+P\right)}^{2}}{2NP}}}{\sqrt{2\pi }(N+P)}$$

We note that, while in the exact expression for *N*_*V*_ (Equation 6) the *P*↔*N* exchange symmetry is lost, in the approximate form the symmetry is recovered.

For sake of comparison with the previous literature, it is also useful to express the main results as a function of α≐*P*/*N*. Equations (4) (for large *N*) and (8) read:

(9) $$p_{B}\approx \frac{1}{2}\left[1-\text{erf}\left(\frac{1+\alpha }{\sqrt{2\alpha }}\right)\right]\;$$

(10) $$N_{V}\approx NP\frac{1}{\sqrt{2\pi }}\frac{\sqrt{\alpha }}{1+\alpha }e^{-\frac{{\left(1+\alpha \right)}^{2}}{2\alpha }}.$$

While *p*_*B*_ only depends on α, *N*_*V*_ clearly is an extensive observable, being proportional to *P* and *N*. Furthermore, both expressions keep their symmetry with respect to the exchange of *P* and *N*, thus to the exchange of α with 1/α. The last observation anticipates that there must exists a region at large α-values where the same features are observed as at small values of α.

### 3.3. Numerical results

To check the predictions of our network model, we have simulated the Model (1) and studied the dynamics for several values of *N* and *P*, in the range of few hundred, see Section 2.3 for details. In the numerical analysis, the *P* memory vectors have been randomly chosen and used to construct the connection matrix **J**. Next, we tested whether or not the stored memories were fixed points of the dynamics. The values of *p*_*B*_, *p*_*V*_ and *N*_*V*_ were calculated by averaging over (up to) 1000 different random realizations of ${\bar{\xi }}^{\mu }$. The results of the numerical simulations are reported (dots) in Figure 1, together with the analytical Expressions (4)–(6) (lines). The three panels refer to the three quantities *p*_*B*_ (Figure 1A), *p*_*V*_ (Figure 1B), and *N*_*V*_ (Figure 1C) as a function of *P* for the selected values of *N*, as reported in the legend. From Figure 1, we observe that on increasing *P*, at fixed *N*, both the single bit probability error, the probability of recovery error (*P*_*V*_), and the number of wrong recoveries *N*_*V*_, after a first fast increase, reach a maximum (equal to 0.02275 for *p*_*B*_, close to one for *p*_*V*_, and larger than *N* for *N*_*V*_) then start to decrease, tending to zero for very large *P*-values.

**Figure 3:**
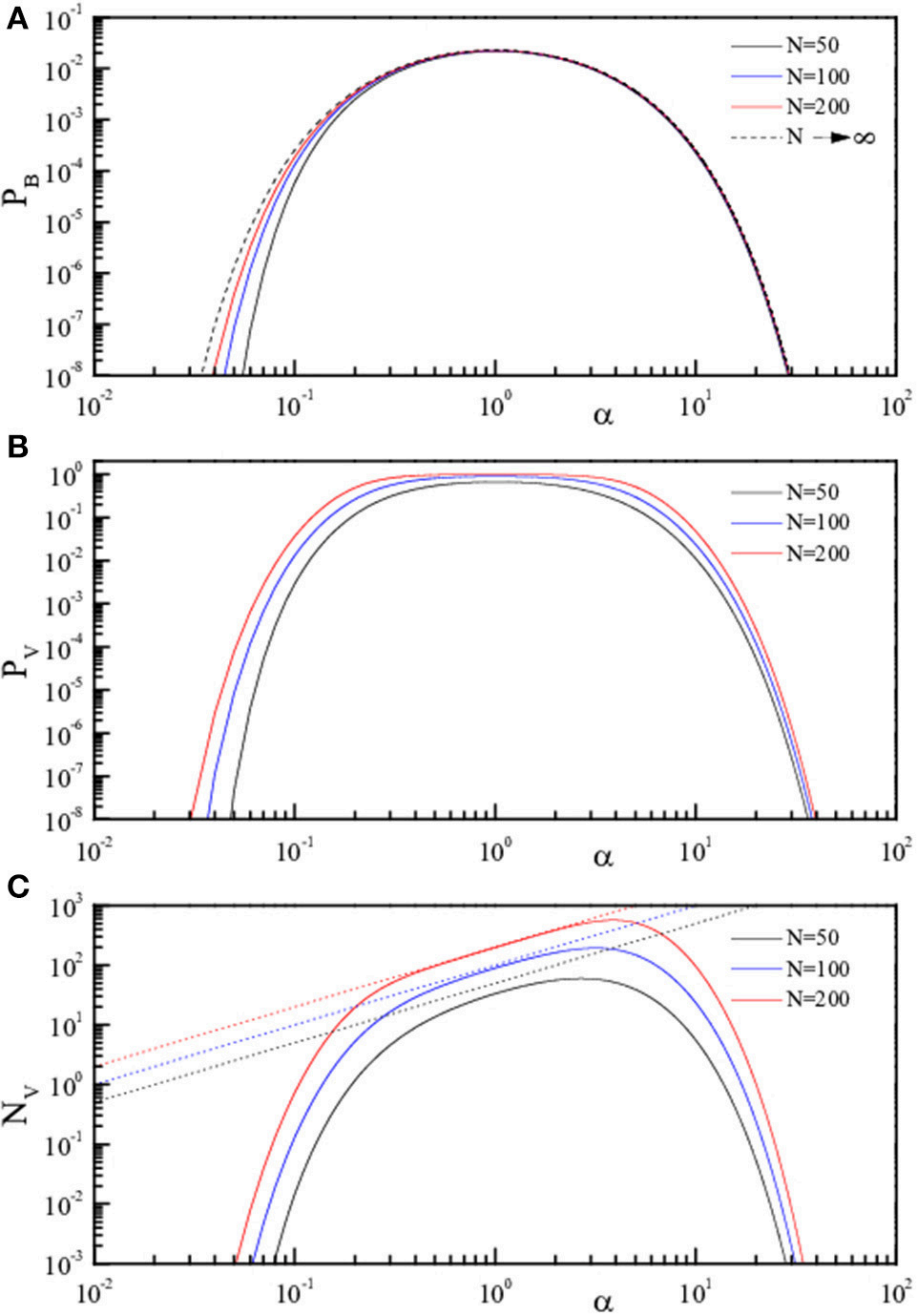
**Theoretical curves for the three quantities *pB, pV*, and *NV* (*pB*, Equation 4, A**; *pV*, Equation 5, **B**; *NV*, Equation 6, **C**) reported in Log-Log scale as full lines. The three quantities are reported in linear scale as a function of α = *P/N* for fixed *N*. The values of *N* are 50 (black), 100 (blue), and 200 (red). The dotted lines in **(C)** represent *NV* = *P*.

To better emphasize this behavior, the same quantities are reported (analytic results only) as a function of α in Figure 2 (linear scale) and in Figure 3 (log scale) for selected *N*. The dotted lines in panels C of both figures represent *N*_*V*_ = *P*, i.e., indicate the case of “totally wrong recovery.” Due to the already observed α↔1/α symmetry, the asymptotic curve in Figure 3A appears with a left-right symmetry around α = 1. From Figures 2, 3, we can clearly identify two regions of high recovery efficiency. The low α region, already studied many years ago by Hopfield (1982); Hopfield et al. (1983); Hopfield (1984) and Amit et al. (1985a,b), shows the existence of a quick transition toward “loss of memory recover” on increasing α around α ≈ 0.14. The second region at large α-values is not yet explored.

Although the value α = 1 (*P* = *N*) represents traditionally a sort of limit in the computation of the storable memories in a RNN, there is no reason why not to store more than *N* memory elements in a network of *N* neurons, that by construction allows 2^*N*^ possible patterns. Indeed, the number of fixed points in a (random) symmetric matrix is known to be, for fully connected symmetric matrices as in our case, exponentially large with *N* (Tanaka and Edwards, 1980). Specifically, the number of fixed points *P*_*o*_ is equal to *P*_*o*_ = exp(γ*N*), with γ ≈ 0.2. *P*_*o*_, much larger than *N*, can be considered a natural limit for *P*.

The recovery efficiency increases for large *P*. In fact, the coherent term in the argument of the sign function increases linearly with *P* and the noise increases as *P*^1/2^. For large *P*, the relative weight of the noise decreases as *P*^−1/2^, this allows to store a large number of memories in a relatively small neural network.

For practical purposes, as for example in the design of an artificial neural network with high efficiency (large storage capacity) and effectiveness (low recovery error rates), it is important to study (Equation 6, and its approximation in Equation 8) and, in particular, to find the conditions for which the network shows “perfect recovery.” Let's define perfect recovery as the state where the number of retrieval errors *N*_*V*_ is smaller than one.

**Figure 4:**
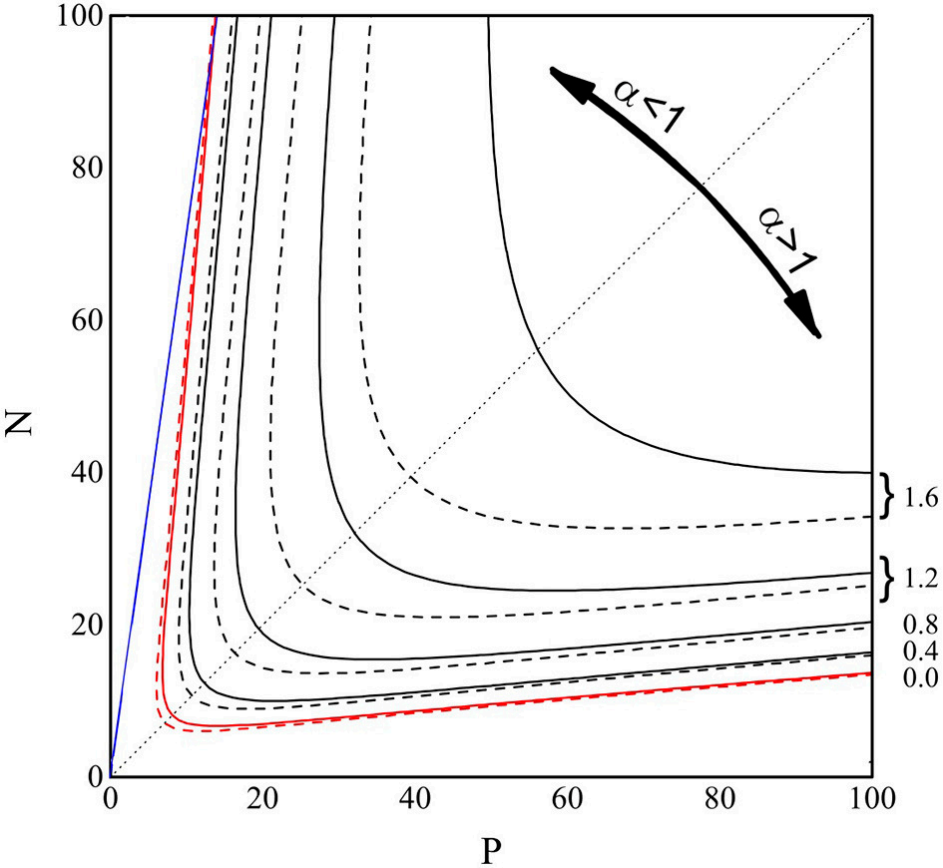
**Contour plot of log_**10**_(***N***_***V***_) from Equations (6) (full lines) and (8) (dashed lines), in the ***P*** and ***N*** range 0–100**. The lines are the loci of the points where *log*_10_(*N*_*V*_) equals 0.0 (red), 0.4, 0.8, 1.2, and 1.6 (black), as indicated on the right side of the figure. The blue line represents *P* = 0.14*N*, while the black dotted line is the bisectrix *N* = *P*, plotted to emphasize the symmetry of the contour lines.

In Figure 4 we show the contour plot of the (decimal) logarithm of *N*_*V*_, from Equations (6) and (8), in the *P*-*N* range [0–100]. The full lines are the loci of the points where *log*_10_(*N*_*V*_) equals 0, 0.4, 0.8, 1.2, and 1.6, as indicated on the right side of the figure. The dashed lines are the same level lines for the (logarithm of the) approximate form of *N*_*V*_ reported in Equation (8). As can be observed, for *N*_*V*_ ≈ 1, the approximation (Equation 8) for *N*_*V*_ is highly accurate, indicating that this approximation can be safely applied to find the “perfect recovery” condition.

In the *P*-*N* plane the existence of two regions (small and large α) where the perfect recovery (*N*_*V*_ = 1, red lines) takes place can be easily observed and the result is symmetric under the exchange of *P* and *N*. In the already explored small α region, we also show (full blue line) the *P* = 0.14*N* condition. Similar to the high α region, it is important to find a simple relation between *N* and *P* identifying the *N*_*V*_ = 1 condition. We aim, therefore, to obtain a function *P*(*N*) which returns, at given *N*, the *P*-value such that *N*_*V*_ = 1. We write the prefactor *NP* in Equation (11) as α*N*^2^ and exploit the α≫1 limit, so to obtain $N_{V}\approx N^{2}\alpha^{1/2}$ exp$\left(-\alpha /2\right)/\sqrt{2\pi }$. The equation *N*^2^α^1/2^exp$\left(-\alpha /2\right)/\sqrt{2\pi }=1$ can be squared, αexp(−α) = 2π/*N*^4^, and solved with respect to α, to give $\alpha =-W_{-1}\left(-2\pi /N^{4}\right)$, where *W*_−1_(*x*) is the second real branch of the Lambert function (Olver et al., 2010). In conclusion, the “perfect recovery condition” is satisfied -for each *N*-value- if we chose to store a number of memories *larger* than *P*(*N*) given by:

(11) $$P(N)=-NW_{-1}(-2\pi /N^{4}).\;$$

For practical purposes, for large enough *N*, we can use the small-argument expansion of the Lambert function −*W*_−1_(−*x*) ≈ −ln(*x*)+ln(−ln(*x*)) (Corless et al., 1996), to have:

(12) $$P(N)=N\left[\text{ln}\left(\frac{N^{4}}{2\pi }\right)+\text{ln}\left(\text{ln}\left(\frac{N^{4}}{2\pi }\right)\right)\right].$$

**Figure 5:**
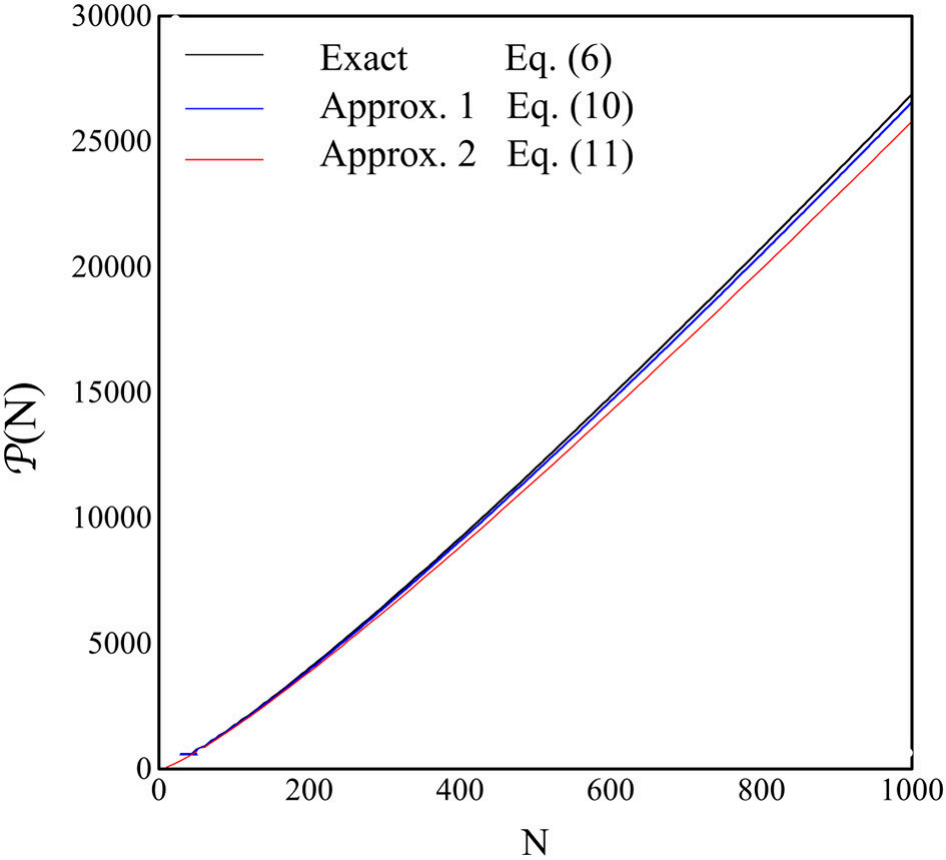
**The quantity ***P***(***N***), i.e., the ***P***-value where the perfect recovery is guaranteed, is shown as a function of ***N*****. The blue line is the numerical solution of *N*_*V*_ = 1 from Equation (6), the blue line is the plot of Equation (12) and the red line is the plot of Equation (13).

The results for *P*(*N*) are shown in Figure 5 as a function of *N* in the range 1–1000. The black line represents the exact, numerical, solution to *N*_*V*_ = 1, with *N*_*V*_ in Equation (6), the blue line is the expression for *P*(*N*) in Equation (11), while the red line is those in Equation (12).

**Figure 6:**
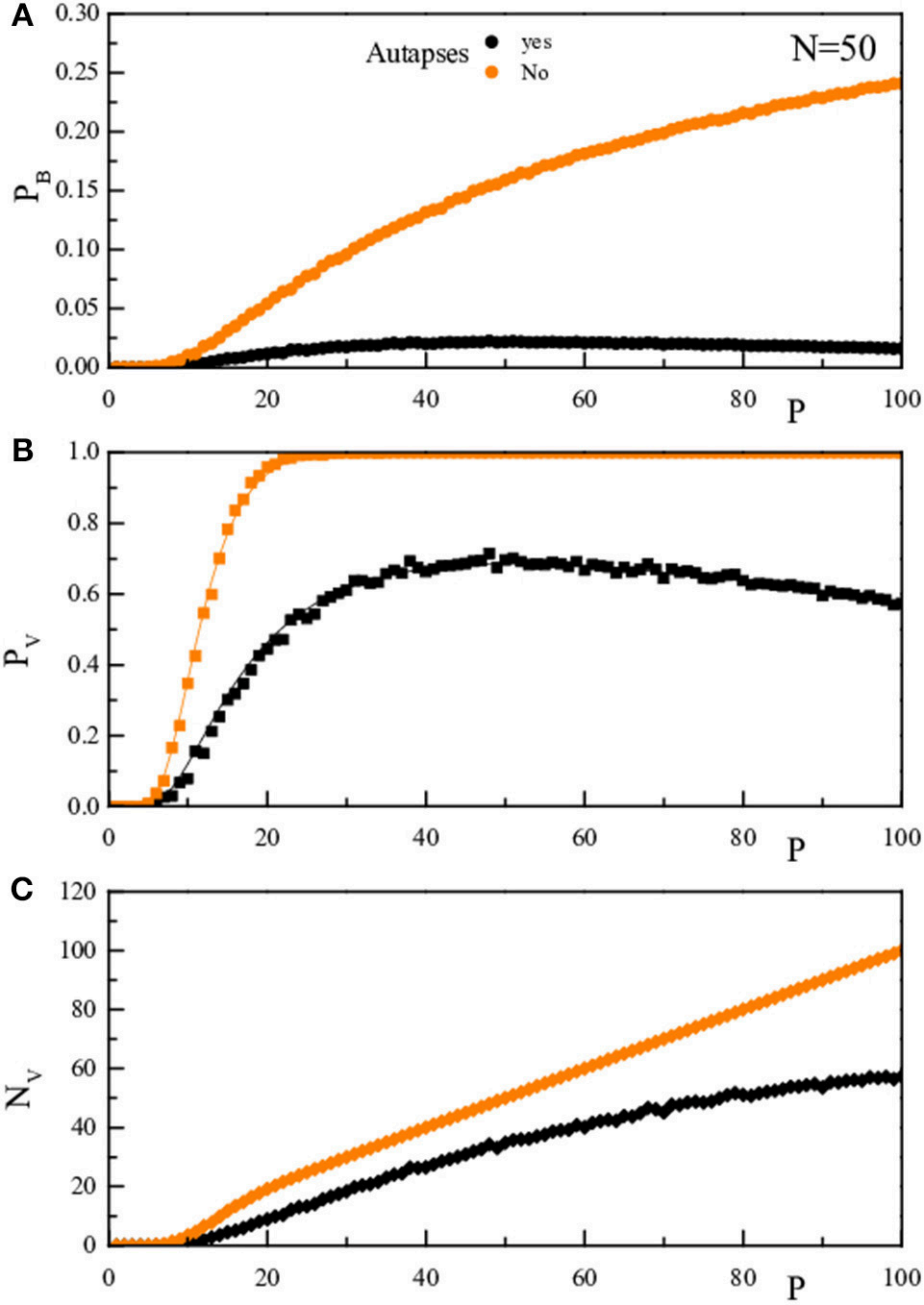
**The upper panel (A) reports for a given ***N***-value (***N*** = 50), as a function of P, the probability ***p***_***B***_ that, stimulating the network with a vector inside the training set, there is one bit wrong in the network response**. The middle panel **(B)** reports *p*_*V*_, the probability that, stimulating the network with a vector inside the training set, the vector obtained after one dynamical step is not the stimulating vector. The lower panel **(C)** reports *N*_*V*_ = *Pp*_*V*_. The black symbols/lines refer to the case where the diagonal elements are as determined in Equation (2), while the oranges ones to diagonal elements forced to vanish. The full lines are the theoretical prediction, the full dots are the results of the numerical simulation.

It is important to note that the presence of a decrease of the retrieval error probability at high *P*, or α, values is due to the presence of non-zero diagonal elements in the *J* matrix that creates a coherent term of weight *P*. Indeed, repeating the rationale leading to Equation (4) with the assumption that *J*_*ii*_ = 0, would give rise to the same (Equations 4–6) but with the numerator of the argument of the error functions equal to *N* − 1 instead of to *N* + *P* − 1. This is shown graphically in Figure 6 where we compare for *N* = 50, both theoretically (full line) and numerically (full dots), the quantities *p*_*B*_, *p*_*V*_, and *N*_*V*_ as a function of *P* in the two cases: diagonal elements in Equation (2) (black) and diagonal elements forced to vanish (orange).

The stabilization of the fixed points ${\bar{\xi }}^{\mu }$ in the high storage region arises from the presence of the non-zero diagonal elements. Asymptotically, on increasing *P*, the diagonal elements growth coherently and the **J** matrix tends to become the unit matrix. However, the dynamics (see Equation 1) dictated by the matrix J does not tend to the dynamics dictated by the unit matrix. In the latter case, indeed, all the 2^*N*^ state vectors should become fixed points and the network should loose on important feature: the capability to distinguish between the stored memories (the vectors ${\bar{\xi }}^{\mu }$, for μ = 1…*P*) and the spurious fixed points, all the vectors $\bar{\zeta }$ not belonging to the set ${\bar{\xi }}^{\mu }$ but such that $E\left[\bar{\zeta }\right]$ = $\bar{\zeta }$. To study this property, we have calculated the probability that a (randomly chosen) vector $\bar{\zeta }$ (different from all the ${\bar{\xi }}^{\mu }$ used to build the *J* matrix) was recognized as a “memory” from the network dynamics. To be consistent with the previous notation (where we called *p*_*B*_ and *p*_*V*_ the probability of errors, not that of correct retrieval of the memory states) we define ${\bar{p}}_{B}$ (${\bar{p}}_{V}$) as the probability of correctly not retrieving a vector not belonging to the training set. More specifically, the quantity ${\bar{p}}_{V}$ is the probability that one dynamical step after presenting a vector ζ not belonging to the training set to the network, the output a vector is different from ζ.” More specifically, the quantity ${\bar{p}}_{V}$ is the probability that presenting a vector ζ not belonging to the training set to the network, after one dynamical step we found as output a vector different from ζ. Similarly for ${\bar{p}}_{B}$. It turns out that ^3^:

(13) $${\bar{p}}_{B}=\frac{1}{2}\left[1-\text{erf}\left(\frac{P}{\sqrt{2(N\text{​}-\text{​}1)(P\text{​}-\text{​}1)}}\right)\right]$$

(14) $${\bar{p}}_{V}=1-{\left[\frac{1}{2}+\frac{1}{2}\text{erf}\left(\frac{P}{\sqrt{2(N\text{​}-\text{​}1)(P\text{​}-\text{​}1)}}\right)\right]}^{N}.$$

**Figure 7:**
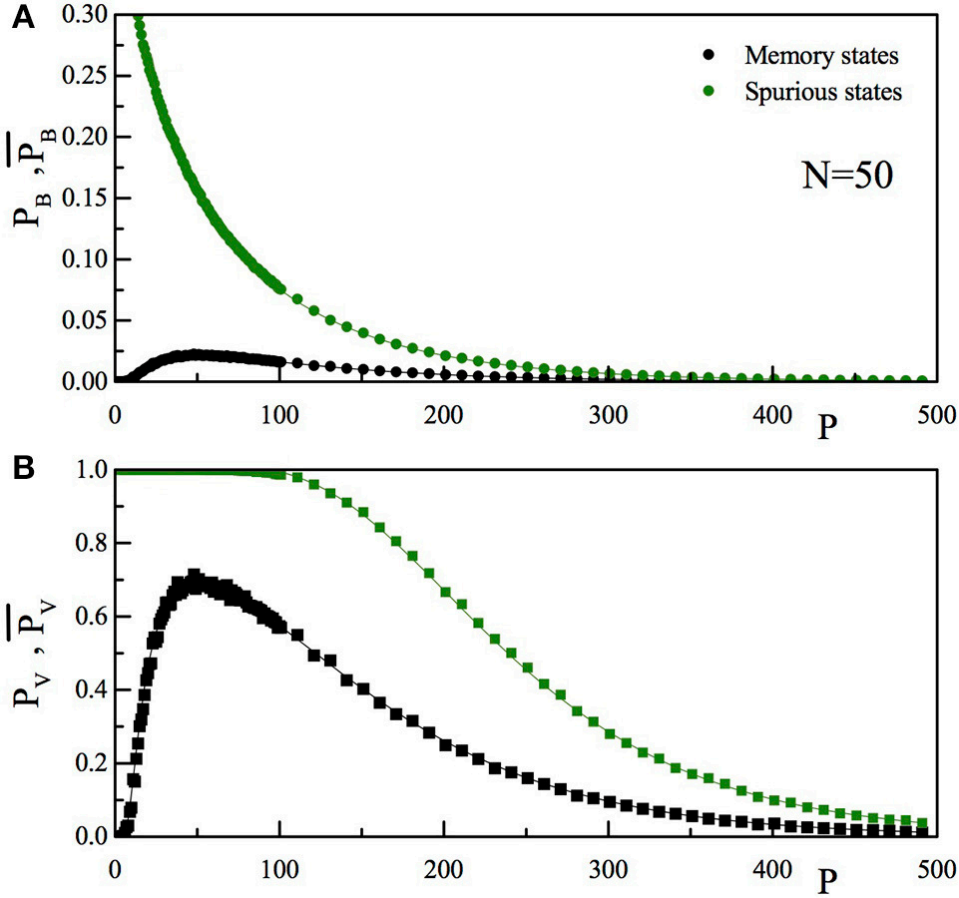
**(A)** The upper panel reports for a given *N*-value (*N* = 50), as a function of P, the probability that, stimulating the network with a vector inside (*p*_*B*_, black) or outside (${\bar{p}}_{B}$, green) the training set, there is one bit differing between the input and the output vector. **(B)** The lower panel reports the probability that, stimulating the network with a vector inside (*p*_*V*_, black) or outside (${\bar{p}}_{V}$, green) the training set, the vector obtained after one dynamical step is not the stimulating vector. The full lines are the theoretical prediction, the full dots are the results of the numerical simulation.

In Figure 7 we report the comparison of the *P* dependence of *p*_*B*_ and ${\bar{p}}_{B}$ (Figure 7A) and that of *p*_*V*_ and ${\bar{p}}_{V}$ (Figure 7B). As usual, full lines are the theoretical results, while the full dots are the outcome of the numerical simulation. Black data are for the “memory states,” while the green ones are for the “spurious state.” As can be seen, the spurious state becomes more and more “present” in the set of memories stored by the network as *P* increases. It seems however that also at high *P*-values the retrieval of the memory states is reasonably good and that of the spurious states reasonably bad.

To be quantitative on this point, we rewrite Equation (14) in its large *N* limit:

(15) $${\bar{p}}_{V}\approx \frac{N^{3/2}P^{-1/2}e^{\;-\;\frac{P}{2N}}}{\sqrt{2\pi }}\;$$

and compare it with Equation (7). In particular, is interesting to calculate the ratio, ρ, between the probability of wrong retrieval of a spurious state and that of a memory state: ρ = ${\bar{p}}_{V}/p_{V}$. From Equations (7) and (15) it turns out:

(16) $$\rho =\left(\frac{N+P}{P}\right)\;e^{\frac{{\left(N+P\right)}^{2}}{2NP}}e^{-\frac{P}{2N}}.$$

This quantity only depends on α:

(17) $$\rho =\left(\frac{1+\alpha }{\alpha }\right)\;e^{\left(1+\frac{1}{2\alpha }\right)}$$

and ρ has a finite high α limit:

(18) $$\underset{\alpha \to \infty }{\lim}\rho =e.$$

In other words, although the number of spurious attractors tends to increase for *P* ≫ *N*, the vectors encoded into the system through the connection matrix are retrieved with an efficiency almost three times better than for the spurious states.

## 4. Discussion

In this work we have developed a simple theoretical approach to investigate the computational properties and the storage capacity of feed-forward networks with self-connections. We have worked out an exact expression which gives the probability *p*_*B*_ of having a wrong bit in the recovery of a memory element from a Hebbian *N*-node neural network, where *P* memory elements are stored. In disagreement with previous studies we have investigated the case in which the diagonal elements were not forced to vanish. Studying the storage capacity, and deriving the related probability *p*_*V*_ and number *N*_*V*_ of having a wrongly recovered memory element, we discovered that besides the well know *P*≪*N* region, there is another region, at *P*≫*N*, where the recovery is highly effective. When *P*≫*N*, the efficiency of recall for a large number of encoded vectors in the *J* matrix is related to the presence of non-zero diagonal elements of the matrix. Basically, the higher storage performance of the network depends on the number of “coherent” terms (the signal) in the quantity $A_{i}^{\mu }$ (see Section 3.1) with respect to the “incoherent” ones (the noise). The larger the ratio between coherent to incoherent terms, the lower the probability of a wrong recovery. The number of coherent terms is (*N* + *P* − 1) in the case of autapses, it is (*N*−1) in the case of no autapses. Indeed, the *P* terms disappear if the diagonal is forced to be zero as in the standard Hopfield model. It is clear that, apart from a transient regime at *P* ~ *N*, increasing *P* ≫ *N* strongly reinforces the signal-to-noise ratio and induces a much larger storage capacity. In addition to the vectors encoded into the system, other unwanted memories also appear in the network. These are the spurious states, fixed points which do not belong to the training set. The presence of spurious states is not a feature specific to the present model, it is a typical characteristic of the standard Hopfield network and its successive improvements. Indeed, as shown by Tanaka and Edwards (1980), a random *N* × *N* matrix has 2^γ*N*^ fixed points (γ ≈ 2). As an example, if *N* = 100, the number of fixed points is about one million. A Hebbian 100 × 100 matrix storing *P* = 1000 patterns, besides the “good” *P* fixed points have also an overwhelming number of spurious fixed points (or “false memories”). The interest of our approach does not rely in “how many” spurious (i.e., not belonging to the training set) states are present but rather in how the recognition of a vector belonging to the training set is as a “good” one. Obviously, the argument of Tanaka-Edwards applies only to random matrices. The Hebbian form, with or without autapses, is not fully random (there exists correlation among the matrix elements), but we expect a number of fixed points similar to that of a random matrix. It would be interesting to determine such a number, but this is beyond the scope of the present paper. In spite of the overwhelming majority of spurious fixed points, the network—even at very large *P*-values, maintains the capacity of discriminate between “good” state (belonging to the training set) and “wrong” ones (not belonging to the training set). More specifically, looking at the one-step dynamical evolution and comparing the input vector with the output one, we have posed to the network the question: “is the input vector belonging to the training set”? We have demonstrated that, when the input vector actually belongs to the training set, at large *P* (similarly to low *P*) the probability of having a wrong response (“no, it does not belong to the training set) goes to zero. Furthermore, we have demonstrated that when the input vector does not belong to the training set the probability of a wrong response (“yes, it is a fixed point”) is much less that in the previous case, asymptotically 2.7 time worst.

In order to identify whether or not a vector belonging to the training set was a fixed point we propose to the system a vector of the training set as input. Then we perform a one-step dynamic evolution of this input state. If after one step the output vector is equal to the input one, this is a fixed point. On the contrary, if after one step the output vector is not equal to the input one, it could be possible that further dynamical steps lead to the input vector. From this point on, as the dynamic is deterministic, the system enters a limit cycle (of length greater than one). Since it is not clear whether or not a limit cycle can be considered a “right recognition,” we have excluded this possibility from the counts of the right recognition. Only fixed point are considered “good.” For this reason, to determine the probability of “right recognition” one dynamical step is enough. We have also not considered the possibility that, using as input a vector not belonging to the training set, it converges to one of the training vectors. The probability of right recognition reported here is an underestimation of the network capability. A further quantity that it would be interesting to evaluate is the size of the attraction basin of a given fixed point, i.e., how many non-training vectors converge to a given training vector fixed point. The basins size would be an important measure of the network performance, their determination is however difficult to achieve analytically, and is behind the goal of the present paper.

One important finding is summarized in Equation (18). It states that for *P* ≫*N*, when the connection matrix is dominated by the diagonal term and is still different from the unity matrix (this is due to the great number of off-diagonal elements with zero average and RMS of the order of $1/\sqrt{P}$), the network retains its capacity of give more “good” than “wrong” answers. This property, the fact that the limit in Equation (18) is *e* and not “1,” can be ascribed to the observation that, although the matrix **J** tends to the unit matrix for large *P*, the dynamics (see Equation 1) dictated by the matrix J does not tend to the dynamics dictated by the unit matrix. This finding opens the way to a much more efficient use of the artificial Hebbian neural network for information storage. In the first region, as well known since 40 years, the storage capacity is limited as the number of encoded vectors becomes of the order *N*. Indeed, in the high α region, the number of elements is basically unlimited ^4^, when the number of stored elements is taken *larger* than ≈ 4*N*ln(*N*).

## Author contributions

GR designed research. VF, ML, and GR performed numerical simulations, analyzed data and wrote the manuscript.

### Conflict of interest statement

The authors declare that the research was conducted in the absence of any commercial or financial relationships that could be construed as a potential conflict of interest.

## Footnotes

^1^The evolution *E*[ξ_*i*_] always return ξ_*i*_ since the sum $\underset{j}{\sum }\xi_{j}^{2}=N$.

^2^These are *P* terms that are present only if the diagonal elements are kept as they are and are not forced to vanish.

^3^The calculation follows the same steps already depicted before, counting the “coherent” terms, that, in this case, only arise from the diagonal elements (*i*=*j*) and not from the μ=ν terms that now do not exist. The weight of the coherent part is equal to *P* instead of *N* + *P* − 1. The rest of the demonstration follows straightforward.

^4^The word unlimited is obviously non-physical. However, the value of *P*_*o*_ arising from the Tanaka-Edwards relation (Tanaka and Edwards, 1980), *P*_*o*_ = exp(γ*N*), with γ = 0.2. *P*_*o*_, already at the *N*-values reported in Equation (5) is so large ($P_{o}\left(N=1000\right)\approx 10^{87}$) with respect to *P*(*N*) (*P*(*N* = 1000) ≈ 210^4^ to state that *P*_*o*_ is unlimited to any practical purpose.
